# AI-driven telerehabilitation for older adults with mild cognitive impairment: a randomized controlled trial

**DOI:** 10.3389/fneur.2026.1813694

**Published:** 2026-04-28

**Authors:** Minsong Kim, Doo Young Kim, Taeksoo Jeong, Si-Woon Park

**Affiliations:** Department of Rehabilitation Medicine, International St Mary's Hospital, Catholic Kwandong University College of Medicine, Incheon, Republic of Korea

**Keywords:** artificial intelligence, cognitive training, Mild Cognitive Impairment, mobile application, telerehabilitation

## Abstract

**Introduction:**

With the rising prevalence of aging populations, accessible interventions for Mild Cognitive Impairment (MCI) are critical. MCI, a high-risk transitional state to dementia, is considered the most opportune window for intervention to maintain or enhance cognitive function. This study evaluates the clinical efficacy of Zenicog^®^, an Artificial Intelligence (AI)-driven, self-guided, home-based cognitive rehabilitation program designed to overcome logistical barriers of conventional therapy.

**Methods:**

A total of 70 older adults (aged 65 years or older) with MCI (indicated by a K-MMSE2 score between 18 and 26) were recruited for this delayed-treatment parallel randomized clinical trial with an exploratory crossover phase. Participants were randomized into Group AB (intervention-first) or Group BA (control-first). The intervention consisted of a 5-week home program (24 sessions) using an AI algorithm for autonomous difficulty adjustment. The primary outcome was global cognitive function, measured by the K-MMSE2. Secondary outcomes included domain-specific cognitive tests [Digit Span Forward (DSF), Digit Span Backward (DSB), Trail Making Test-A/B (TMT-A/B)], psychosocial measures and usability and adverse events were also assessed. Primary analysis focused on Period 1 (T1), comparing Group AB (intervention) to Group BA (control). Participants were randomized via a computer-generated block randomization method. Randomization used concealed allocation via sequentially numbered opaque envelopes prepared independently, with group assignment performed by a blinded investigator.

**Results:**

Sixty-two participants were included in the final analysis, randomly allocated to Group AB (*n* = 35) or Group BA (*n* = 35). At the end of Period 1, the intervention group showed significantly higher K-MMSE2 scores compared to the control group (median 28.0 vs. 26.0; *p* < 0.001). Crossover analysis confirmed significant K-MMSE2 gains occurred only during the intervention periods for both groups. Clinical success (K-MMSE2 ≥27) was achieved by 93.9% of the intervention group vs. 0% of the control. No significant differences were found in domain-specific tests. Usability and satisfaction scores were high (≥4.5/5), with zero dropouts due to adverse effects.

**Discussion:**

AI-driven, self-guided telerehabilitation is a feasible and effective strategy for improving global cognitive function in MCI patients. Its scalability and minimal supervision requirements make it a viable alternative to clinic-based therapy in aging societies.

**Clinical Trial Registration:**

Clinical Research Information Service (CRIS), KCT0008968, https://cris.nih.go.kr/cris/search/detailSearch.do?seq=31040&search_page=L. Registered on November 21, 2023.

## 1 Introduction

The global demographic transition toward an aging population poses a growing public health challenge, particularly in countries undergoing rapid shifts, such as South Korea. South Korea transitioned into an aged society (proportion of population aged 65 years and older exceeding 14%) in 2018 and further became an ultra-aged society (over 20%) in 2025 (1). This demographic trend is closely associated to the increasing prevalence of age-related cognitive disorders, which severely diminish independence in performing activities of daily living (ADLs) and negatively impact overall quality of life (2, 3).

Systematic reviews suggest that while Mild Cognitive Impairment (MCI) does not cause significant functional impairment, it is associated with a noticeable decline in health-related Quality of Life (QoL) compared to healthy older adults, underscoring the need for timely intervention (2). A critical area of focus is MCI, which represents a transitional state between normal aging and dementia, with high annual conversion rates to Alzheimer's disease and other forms of dementia. Specifically, the annual progression rate from MCI to dementia is estimated to be approximately 10%−15%, with a cumulative risk reaching 30%−40% over 5 years (4, 5).

Given this trajectory, the MCI stage is considered the most opportune window for intervention, aiming to maintain or enhance cognitive function and delay the progression to dementia (6). Cognitive Rehabilitation (CR), which targets core cognitive domains such as memory, attention, and executive function, has therefore become an essential therapeutic strategy for the management of MCI, demonstrating positive effects on cognitive function in multiple systematic reviews and clinical trials (6, 7).

To meet the surging demand for CR in this large and growing population, computerized cognitive rehabilitation, defined as the delivery of cognitive training via digital platforms, has emerged as an increasingly adopted therapeutic modality, offering standardized, repeatable, and objective intervention (8, 9). Computerized cognitive rehabilitation has been widely adopted across various countries and has shown promising effects in maintaining or improving cognitive performance and supporting independence (9).

However, the successful long-term application of computerized cognitive rehabilitation for the elderly and frail MCI population faces significant clinical and logistical barriers. Logistically, older adults and patients with physical frailty often face substantial challenges in traveling to clinics or hospitals for regular, in-person computerized cognitive rehabilitation sessions, leading to poor adherence and premature discontinuation of therapy. Clinically, while traditional computerized cognitive rehabilitation systems offer self-guided learning, they typically rely on fixed or sequential task difficulty, necessitating constant therapist intervention to dynamically adjust the training load based on performance, and to reliably monitor patient engagement in unsupervised home environments. These limitations severely restrict the scalability of computerized cognitive rehabilitation as a continuous, home-based care solution.

To overcome these constraints and realize the full potential of telerehabilitation, defined as the remote delivery of rehabilitation services to a patient's home or community, advanced platforms integrating cloud computing and Artificial Intelligence (AI) have recently emerged. Zenicog^®^ (Mindhub Inc., Anyang, Republic of Korea) is an AI-driven telerehabilitation system, characterized by its capacity to autonomously optimize training through real-time data analysis. Using cloud-based infrastructure, it monitors performance metrics—such as accuracy, response time, and completion rate—to dynamically adjust task complexity, ensuring personalized intervention without continuous human oversight (10).

Despite the promise of such autonomous systems, the clinical efficacy and feasibility of self-guided, AI-driven telerehabilitation—especially one designed to function with minimal human intervention—have not been rigorously evaluated in the target MCI population. Existing studies largely focus on therapist-supervised remote models or fixed training protocols, leaving a critical gap regarding the effectiveness and adoption of truly independent digital solutions. This study aims to evaluate the clinical effectiveness of a self-guided, AI-driven telerehabilitation program (Zenicog^®^) for patients with mild cognitive impairment. The findings are expected to provide empirical evidence supporting the integration of autonomous digital health technologies into community-based cognitive care.

## 2 Materials and methods

### 2.1 Materials

#### 2.1.1 Participants

Participants were eligible for enrollment if they were older adults aged 65 years or older and demonstrated cognitive impairment, as indicated by a Korean version of Mini-Mental State Examination-2 (K-MMSE2) score between 18 and 26. Exclusion criteria included individuals with significant visual, auditory, or physical impairments that could interfere with the use of computerized devices, as well as those with insufficient digital literacy, defined as difficulty independently operating smart devices. Participants with a diagnosis of severe psychiatric disorders, including schizophrenia, dissociative disorders, or major depressive disorders (severe or secondary depression), were also excluded. In addition, the use of medications that could influence cognitive function served as an exclusion criterion. Specifically, patients taking anti-dementia agents such as acetylcholinesterase inhibitors (donepezil, galantamine, rivastigmine) or glutamate-modulating agents (memantine), central nervous system–acting drugs such as antipsychotics, antidepressants, anti-alcohol medications, sedatives, or tranquilizers (including benzodiazepines), and opioid analgesics (e.g., oxycodone) were excluded. All participants provided written informed consent approved by the Institutional Review Board prior to enrollment.

#### 2.1.2 Intervention

The intervention consisted of a 5-week, home-based cognitive training program delivered via a tablet PC using Zenicog^®^ software. The program included 24 sessions, each lasting about 30 minutes, targeting attention, memory, and executive function.

**Figure 1:**
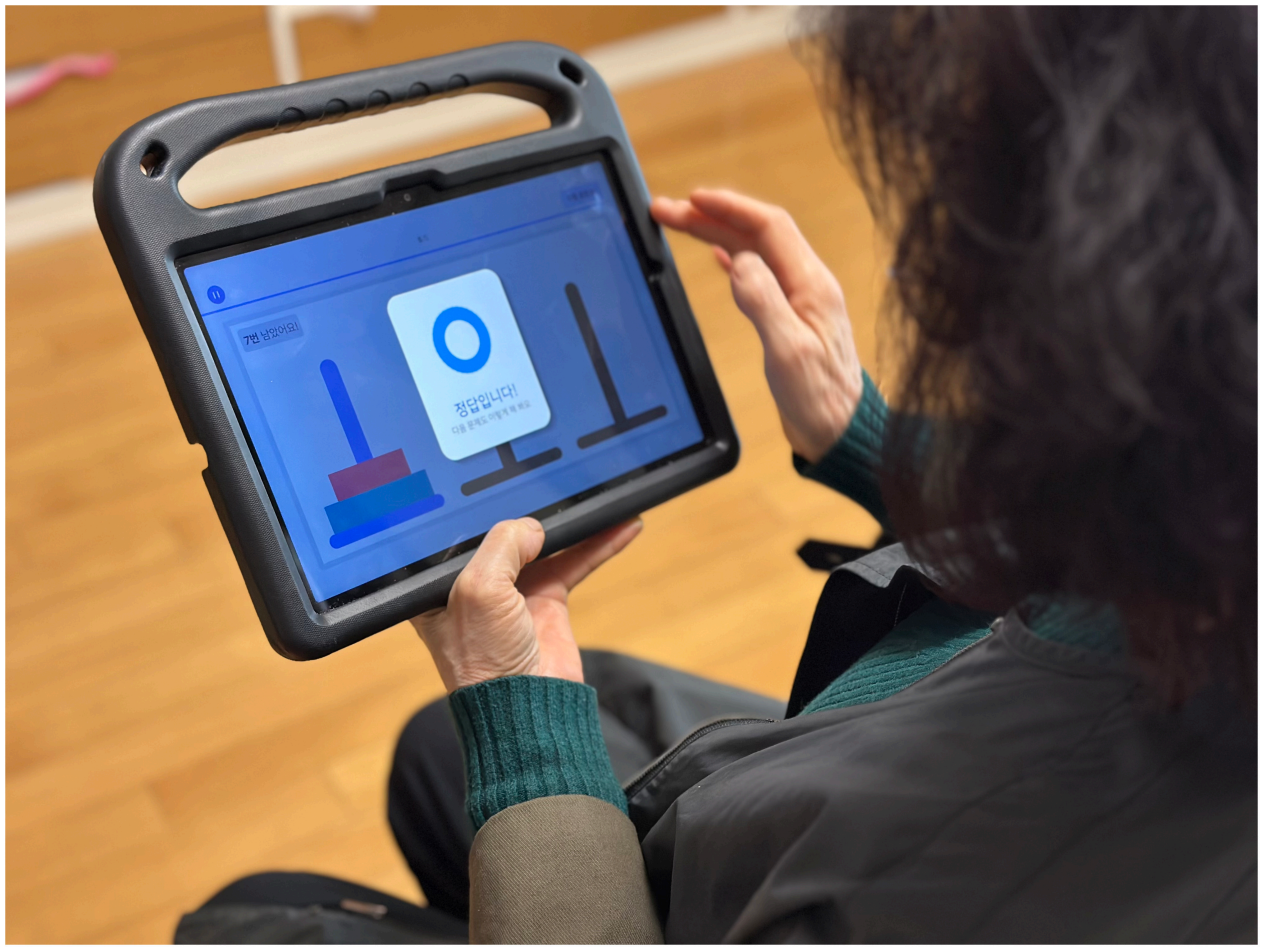
Representative image of a participant utilizing the Zenicog^®^ system.

Zenicog^®^ is an AI-driven computerized cognitive rehabilitation program equipped with adaptive difficulty algorithms, personalized content recommendations, and cloud-based compliance monitoring. The system provides a total of 61 training contents categorized into seven cognitive domains: Attention, Memory, Executive Function, Auditory Comprehension, Speaking, Reading, and Writing. A detailed inventory of these specific training modules and tasks is provided in Supplementary material 1. These technologies enabled individualized cognitive telerehabilitation tailored to older adults with MCI. A representative image of a participant utilizing the Zenicog^®^ system in a home environment is presented in Figure 1, illustrating the user interface and the practical application of the telerehabilitation program. The AI-driven algorithms and cloud-based monitoring systems of Zenicog^®^ utilized in this trial are consistent with those described in our earlier study (10).

The AI-assisted training platform used in this study consisted of four integrated components.

First, the training record module automatically logged key performance indicators for each session, such as accuracy and response time, to monitor individual progress over time.

Second, the task analysis module utilized a predefined database of training items that were organized according to difficulty level and allotted time. By comparing these task characteristics with each participant's recent performance profile, the module facilitated the selection of tasks that aligned with the participant's current abilities.

Third, the performance prediction module generated estimates of upcoming task performance based on patterns observed in previous training sessions.

Finally, the recommendation module synthesized these outputs and applied an optimization procedure to determine the most appropriate tasks for subsequent training, continuously adjusting task selection in response to real-time performance.

The recommendation algorithm implemented in this system is not based on large-scale datasets or deep learning techniques. Instead, it operates as a data-driven adaptive architecture that utilizes multi-criteria scoring and probabilistic decision-making to provide individualized task recommendations based on real-time performance data. The algorithm considers multiple parameters—including accuracy, response latency, item difficulty, time limits, and number of attempts—to evaluate task-specific performance and predict immediate outcomes. These predictions guide the dynamic allocation of forthcoming tasks, enabling rapid adaptation of training intensity and content without the need for hyperparameter tuning or extensive model training. Detailed descriptions of the Zenicog^®^ system and its architecture are provided in Supplementary material 2.

Although formal external validation and generalizability testing have not yet been conducted, preliminary clinical impressions and user feedback suggest that the system is feasible to implement and offers practical utility in cognitive rehabilitation. By automatically adjusting training demands to match participants' evolving cognitive status, the platform supports continuous, personalized training without direct therapist involvement.

Throughout the treatment phase, participants conducted the training independently and without clinical supervision. During the non-treatment phase, participants engaged in their usual routines without formal cognitive training. All assessments were performed at baseline (T0), post-Period 1 (T1), and post-Period 2 (T2).

### 2.2 Methods

#### 2.2.1 Study design

This study employed a delayed-treatment parallel randomized clinical trial with an exploratory crossover phase to evaluate the effectiveness of cognitive telerehabilitation in individuals with MCI. Participants were randomly assigned to one of two groups.

• Group AB: received the telerehabilitation intervention during the first 5-week period (T0 to T1), followed by no treatment (T1 to T2).

• Group BA: underwent no treatment initially, followed by the intervention in the second period.

A crossover design was selected to address both ethical concerns and practical barriers to participant retention. MCI participants are often motivated to improve or maintain cognitive function, and allocation to a control group that received no treatment while undergoing multiple assessments could reduce motivation and increase the likelihood of dropout. The crossover design ensured that all participants eventually received the active intervention, which was expected to improve adherence.

Although the study used a crossover structure, the primary analysis focused only on Period 1 (T0 to T1), where Group AB served as the intervention group and Group BA as the control group. This analytic strategy satisfied the assumptions of a parallel-group randomized controlled trial and reduced the risk of carry-over or time-related confounding. Data from Period 2 (T1 to T2) were used for secondary, exploratory analyses only.

#### 2.2.2 Randomization and allocation concealment

Participants were randomized via a computer-generated block randomization method (https://www.randomizer.org). An independent researcher placed allocation results in sequentially numbered opaque envelopes, which were then opened by a separate blinded investigator responsible for group assignment: the randomization and allocation process were designed to minimize bias and maintain allocation concealment.

#### 2.2.3 Sample size calculation

Sample size was determined using G^*^Power (Version 3.1.9.7), based on an effect size of 0.74 derived from a previous meta-analysis evaluating cognitive training efficacy in older adults (11). With α = 0.05 and power = 0.80, a total sample size of 60 was calculated. To account for potential attrition, 70 participants were recruited. No interim analysis was performed in this study.

#### 2.2.4 Outcome measures

##### 2.2.4.1 Primary outcomes: global cognitive function

Cognitive and functional outcomes were assessed using standardized instruments administered in person by trained staff.

Global cognitive function was measured using the K-MMSE2, a 30-point tool in which higher scores indicate better cognitive performance.

##### 2.2.4.2 Secondary outcomes: domain-specific cognitive tests, psychosocial well-being, functional independence

Attention and working memory were assessed using Digit Span Forward (DSF) and Digit Span Backward (DSB), administered via a computerized neurocognitive test (CNT). Higher scores indicate better function.

Executive function and processing speed were measured with Trail Making Test A and B (TMT-A, TMT-B), also via CNT. These are timed tests; longer times reflect poorer performance. Tests were capped at 180 s (A) and 300 s (B), with non-completers assigned maximum scores.

Depressive symptoms were evaluated with the Korean version of the Center for Epidemiological Studies Depression Scale-Revised (K-CESD-R), a 20-item scale rated on a 0–4 Likert scale, yielding a total score of 0–80, with higher scores reflecting more severe depressive symptoms.

Quality of life was assessed with the EQ-5D index (range 0–1), where higher scores indicate better health-related quality of life. Functional independence was measured with the Seoul-Instrumental Activities of Daily Living (S-IADL), a 15-item tool scored on a 0–3 Likert scale. Higher scores indicate greater impairment.

Self-efficacy was assessed with the Self-Efficacy Scale (SES), a 10-item scale scored 1–4 per item; higher scores indicate greater perceived self-efficacy.

At baseline, the following additional data were collected: age, sex, years of education, Charlson Comorbidity Index (CCI), medical history, current medications, smoking status, and alcohol use.

##### 2.2.4.3 Usability and adverse event

System usability was assessed using a 25-item instrument developed in accordance with Universal Design principles proposed by North Carolina State University to evaluate the accessibility and usability of the remote rehabilitation program (12). The measure encompassed key domains, including equitable and flexible use, simplicity and intuitiveness of operation, perceptibility of information, tolerance for error, minimal physical effort, and adequacy of size and spatial configuration, in addition to overall product quality and global user satisfaction. Each item was rated on a 5-point Likert scale, with higher scores denoting superior perceived usability. The final survey items were refined and validated through a consensus process using the modified Delphi method. Details regarding usability were previously described in depth in our study on feasibility (13).

Participants were informed in advance about potential adverse events, including fatigue and dry eye, and about the planned management strategies, such as recommending rest for fatigue and providing artificial tears for dry eye, if required. These adverse events were prespecified and assessed non-systematically based on spontaneous reports obtained during regular interviews conducted by the research team. No serious intervention-related adverse events or unexpected adverse events were reported during the study. Safety monitoring was maintained after completion of data collection until August 11, 2025, to identify any delayed adverse effects.

#### 2.2.5 Statistical analysis

All statistical analyses were conducted using SPSS version 22.0 (IBM Corp., Armonk, NY, USA), and R version 4.3.2 (The R Foundation for Statistical Computing, Vienna, Austria). Statistical significance was set at *p* < 0.05 (two-tailed). Normality of continuous variables was assessed using the Kolmogorov–Smirnov test. Except for age, all continuous variables violated the assumption of normality. Accordingly, independent two-sample comparisons were conducted using the independent *t*-test for normally distributed variables and the Mann–Whitney *U*-test for non-normally distributed variables. For categorical variables, between-group differences were analyzed using the chi-square test or Fisher's exact test, as appropriate.

The primary analysis was conducted by treating the first phase of the crossover trial (Period 1) as a parallel-group randomized controlled trial (RCT). Mann–Whitney *U*-test were used to compare outcomes between Group AB (who received the intervention during Period 1) and Group BA (who served as the control during the same period). These comparisons were made at both baseline (T0) and post-treatment (T1) to evaluate the between-group effects of the intervention during Period 1.

To account for the repeated-measures structure and the crossover design across all time points (T0, T1, T2), Generalized Estimating Equations (GEE) with an exchangeable working correlation matrix were implemented using the ‘geepack' package in R. The GEE model evaluated the independent effect of intervention status (Active vs. Control) while adjusting for period and sequence effects as covariates to account for potential baseline imbalances and order effects. To control the inflation of Type I error due to multiple outcome measures, p-values for all secondary outcomes were adjusted using the False Discovery Rate (FDR) method in R.

To examine within-subject changes in MMSE scores across the three assessment points (T0, T1, and T2), we employed the Friedman test, a nonparametric alternative to repeated-measures ANOVA. A significant result on the Friedman test indicated that at least one time point differed significantly from the others. *Post-hoc* pairwise comparisons between time points were conducted using Wilcoxon signed-rank tests. These analyses were repeated separately for each sequence group (AB and BA) to investigate whether MMSE gains were temporally aligned with the intervention periods.

Additionally, we explored the clinical relevance of cognitive improvement by categorizing MMSE scores using a cutoff of 27 points, where scores of 27 or higher were defined as cognitive success. This threshold was selected based on recent clinical validation studies in the Korean population, which established an MMSE score of 26 or below as a reliable cutoff for screening MCI (14, 15). Consequently, achieving a score of 27 or higher signifies a return to the cognitively normal range for this demographic. The proportion of participants reaching this threshold at T1 and T2 was compared across groups using chi-square tests.

#### 2.2.6 Ethical considerations

The protocol received Institutional Review Board (IRB) approval from Catholic Kwandong University International St. Mary's Hospital (IS23OISE0063). The study protocol was also registered with the Clinical Research Information Service (CRIS, https://cris.nih.go.kr/cris/index/index.do) prior to participant enrollment (Registration No. KCT0008968). Written informed consent was obtained from all participants before enrollment. The consent process was conducted in a private setting by a physician investigator, ensuring that participants had adequate time to review and understand the study procedures, potential risks, and benefits. For participants with difficulty understanding the consent materials, both the participant and their legal representative received concurrent explanations before written consent was obtained.

## 3 Results

### 3.1 Participant characteristics

**Figure 2:**
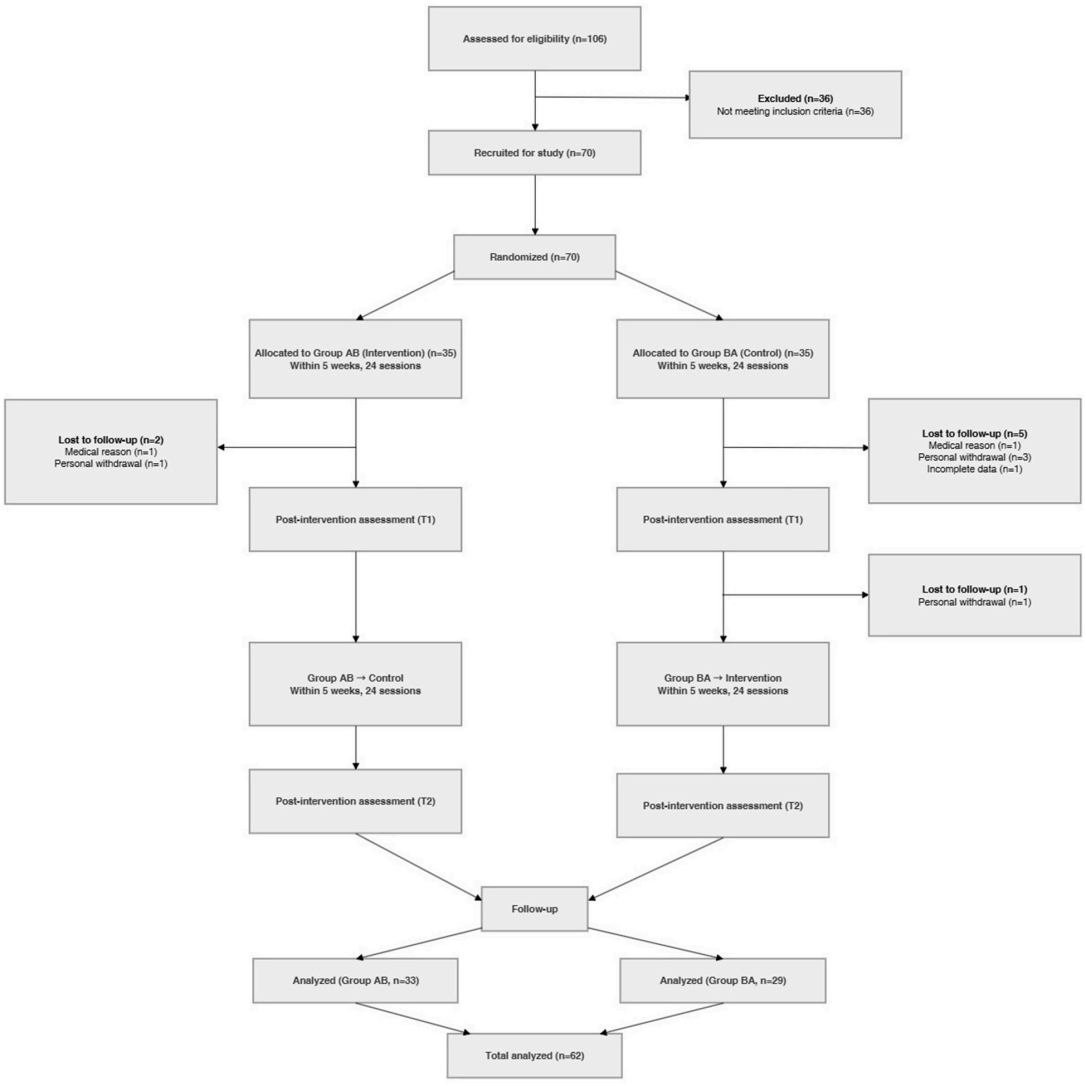
Overview of study design and CONSORT flow diagram.

Participant recruitment commenced on November 22, 2023, and data collection for main outcomes was finalized by June 20, 2025. To ensure a rigorous assessment of safety, including delayed adverse effects, a follow-up monitoring period was maintained until the formal study termination on August 11, 2025. A total of 70 participants were recruited, with 35 allocated to each sequence group (AB and BA). After accounting for dropout (*n* = 2 in Group AB, *n* = 6 in Group BA), 62 participants (*n* = 33 in Group AB and *n* = 29 in Group BA) completed the study (Figure 2). A total of 8 participants did not complete the study. These discontinuations occurred due to personal voluntary withdrawal, medical conditions (e.g., renal failure, surgery), or incomplete outcome data. It is noteworthy that none of these medical events or personal withdrawals were deemed related to the study device or intervention. Consequently, no participant dropout was attributable to device-related adverse effects or complications. The remaining 62 participants (AB group: *n* = 33; BA group: *n* = 29) successfully completed all study procedures and were included in the final analysis.

The overall mean age was 74.0 ± 4.8 years, and 87.1% of participants were female. There were no statistically significant differences between groups in demographic or baseline clinical characteristics, including age, sex, education, CCI, smoking status, and alcohol use.

Similarly, no significant baseline differences were observed between groups in any of the outcome variables, including MMSE, DSF, DSB, TMT-A, TMT-B, CES-D, EQ-5D, and SES (Table 1). While a statistically significant group difference was noted in baseline S-IADL scores (*p* = 0.026), the distribution of S-IADL scores was highly skewed, with the majority of participants scoring 0 and very few reporting values above 2. Given that the total score range of the S-IADL instrument is 0 to 45, this narrow range of responses likely reflects a floor effect, and the observed statistical difference is unlikely to carry meaningful clinical implications.

**Table 1:** Baseline demographic and clinical characteristics of participants.

Characteristics	Total (*n* = 62)	Group AB (*n* = 33)	Group BA (*n* = 29)	*p*-value
Age, yr	74.0 ± 4.8	73.8 ± 4.4	74.2 ± 5.3	0.718
Sex, *n*				0.570
- Male	8 (12.9%)	4 (12.1%)	4 (13.8%)	
- Female	54 (87.1%)	29 (87.9%)	25 (86.2%)	
Education, yr	7.6 ± 3.9	6 [6, 12]	6 [5, 9]	0.265
CCI	0.8 ± 1.0	1.0 [1.0, 1.0]	1.0 [1.0, 1.0]	0.481
Smoking				0.562
- Current smoker	0 (0%)	0 (0%)	0 (0%)	
- Ex-smoker	5 (8.1%)	3 (9.1%)	2 (6.9%)	
- Never	57 (91.9%)	30 (90.9%)	27 (93.1%)	
Alcohol history				0.282
- Heavy	0 (0%)	0 (0%)	0 (0%)	
- Social	8 (12.9%)	3 (9.1%)	5 (17.2%)	
- Never	54 (87.1%)	30 (90.9%)	24 (82.8%)	
MMSE (T0)	26.0 [25.0, 26.0]	26.0 [25.0, 26.0]	26.0 [25.0, 26.0]	0.226
DSF (T0)	5.1 [4.2, 6.1]	5.1 [4.2, 6.1]	5.1 [4.3, 5.3]	0.513
DSB (T0)	3.3 [3.1, 4.1]	3.3 [3.1, 4.1]	3.3 [3.1, 4.1]	0.925
TMT-A (T0)	40.5 [31.25, 53.75]	42.0 [35.0, 54.0]	34.0 [30.0, 50.0]	0.111
TMT-B (T0)	105.5 [77.25, 166.25]	108.0 [78.0, 171.0]	103.0 [77.0, 162.0]	0.494
CES-D (T0)	5.5 [2.0, 14.0]	5.0 [1.0, 18.0]	6.0 [3.0, 13.0]	0.837
EQ-5D (T0)	0.81 [0.76, 0.85]	0.81 [0.75, 0.85]	0.83 [0.78, 0.86]	0.252
SES (T0)	29.0 [27.0, 32.0]	29.0 [27.0, 32.0]	29.0 [27.0, 31.0]	0.972
S-IADL (T0)	2.0 [1.0, 2.75]	2.0 [1.0, 3.0]	1.0 [0.0, 2.0]	0.026^*^

Values are presented as mean ± SD or number (%) or median [1st quartile, 3rd quartile]. MMSE, Mini-Mental State Examination; CCI, Charlson Comorbidity Index; DSF, Digit Span Forward; DSB, Digit Span Backward; TMT, Trail Making Test; CES-D, Center for Epidemiologic Studies Depression Scale; EQ-5D, EuroQoL 5-Dimension; SES, Self-Efficacy Scale; S-IADL, Seoul-Instrumental Activities of Daily Living.

Group AB = Intervention first, then no treatment; Group BA = No treatment first, then intervention. ^*^Indicates statistical significance (*p* < 0.05).

### 3.2 Outcome analysis

At the end of Period 1 (T1), a Mann–Whitney *U*-test revealed that Group AB (who received the intervention) showed significantly higher MMSE scores than Group BA (who received no treatment) [median (1st quartile, 3rd quartile): 28.0 (27.0, 29.0) vs. 26.0 (26.0, 26.0); *p* < 0.001; Table 2]. No other cognitive or functional outcomes showed statistically significant between-group differences at T1.

**Table 2:** Between-group comparison of cognitive and functional outcomes at t1 (end of period 1).

Outcome measures	Group AB (intervention, *n* = 33)	Group BA (no treatment*, n* = 29)	*p*-value	Adjusted *p*-value (FDR)
MMSE (T1)	28.0 [27.0, 29.0]	26.0 [26.0, 26.0]	<0.001	<0.001^*^
DSF (T1)	5.2 [5.1, 6.1]	5.2 [4.2, 6.2]	0.271	0.488
DSB (T1)	3.3 [3.2, 4.1]	4.1 [3.1, 4.1]	0.687	0.778
TMT-A (T1)	33.0 [31.0, 46.0]	35.0 [28.0, 44.0]	0.778	0.778
TMT-B (T1)	86.0 [67.0, 130.0]	110.0 [76.0, 146.0]	0.242	0.488
CES-D (T1)	4.0 [1.0, 8.0]	5.0 [2.0, 9.0]	0.755	0.778
EQ-5D (T1)	0.82 [0.80, 0.86]	0.86 [0.82, 0.86]	0.396	0.594
SES (T1)	29.0 [27.0, 30.0]	30.0 [30.0, 34.0]	0.025	0.113
S-IADL (T1)	2.0 [1.0, 2.0]	1.0 [0.0, 2.0]	0.056	0.168

Values are presented as median [1st quartile, 3rd quartile]. Adjusted *p*-value (FDR) denotes *p*-values corrected using the False Discovery Rate (FDR) method to account for multiple comparisons across all outcome measures.

Group AB received the intervention during Period 1 (T0–T1); Group BA received no treatment during the same period. MMSE, Mini-Mental State Examination; DSF, Digit Span Forward; DSB, Digit Span Backward; TMT, Trail Making Test; CES-D, Center for Epidemiologic Studies Depression Scale; EQ-5D, EuroQoL 5-Dimension; SES, Self-Efficacy Scale; S-IADL, Seoul-Instrumental Activities of Daily Living. ^*^Indicates statistical significance (*p* < 0.05).

**Figure 3:**
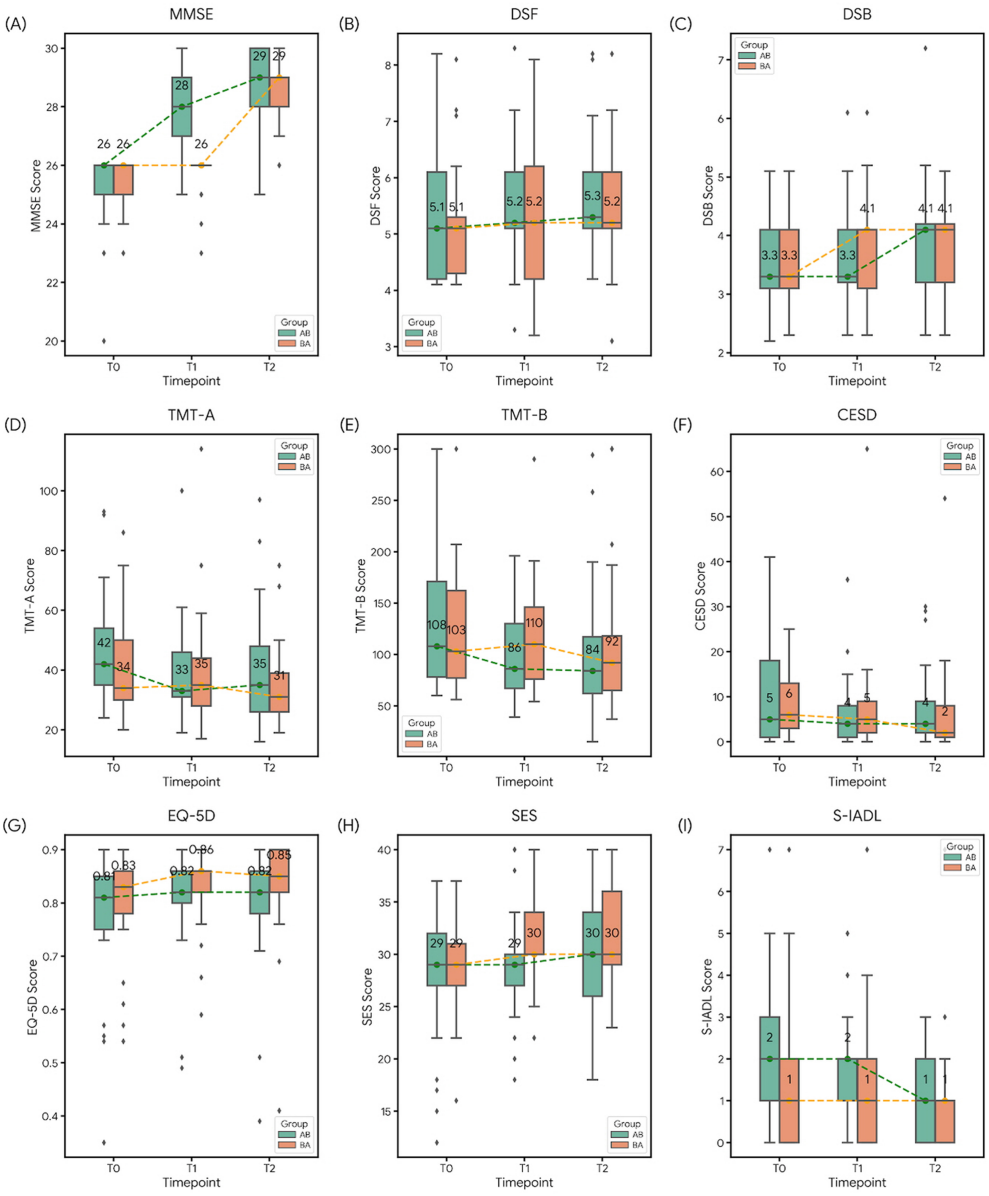
Comparison of cognitive, psychological, and functional outcomes between AB and BA groups across three time-points (T0, T1, and T2).; Longitudinal changes are presented for **(A)** MMSE, **(B)** DSF, **(C)** DSB, **(D)** TMT-A, **(E)** TMT-B, **(F)** CESD, **(G)** EQ-5D, **(H)** SES, and **(I)** S-IADL. In each box plot, the central line indicates the median, the box represents the interquartile range (IQR), and whiskers extend to 1.5 times the IQR. Outliers are represented by individual points. Median values for each group are connected by dashed lines (green for Group AB, orange for Group BA) to illustrate trends over time. MMSE, Mini-Mental State Examination; DSF, Digit Span Forward; DSB, Digit Span Backward; TMT, Trail Making Test; CES-D, Center for Epidemiologic Studies Depression Scale; EQ-5D, EuroQoL 5-Dimension; SES, Self-Efficacy Scale; S-IADL, Seoul-Instrumental Activities of Daily Living.

Descriptive statistics for all outcome measures across the three time points (T0, T1, and T2) by group are presented in Supplementary material 3. A gradual improvement in MMSE was observed in both groups following the intervention period. Other measures showed smaller or inconsistent changes across periods (Figure 3).

The Friedman test conducted across all participants revealed a significant change in MMSE over time (*p* < 0.001). *Post-hoc* Wilcoxon signed-rank tests confirmed that MMSE scores improved significantly from T0 to T1, from T1 to T2, and from T0 to T2 (all *p* < 0.001). Group-specific analyses provided further support for the temporal alignment of cognitive improvement with the intervention period. In Group AB (*n* = 33), who received the intervention during Period 1, MMSE scores improved significantly from T0 to T1 (Z = −5.07, *p* < 0.001) and from T0 to T2 (Z = –5.06, *p* < 0.001). However, no significant change was observed from T1 to T2 (Z = –1.81, p = 0.070), suggesting a plateau following initial gains. In contrast, Group BA (*n* = 29), who received the intervention during Period 2, showed no significant change between T0 and T1 (Z = –1.63, p = 0.102), but demonstrated significant gains from T1 to T2 (*Z* = –4.61, *p* < 0.001) and from T0 to T2 (*Z* = –4.77, *p* < 0.001). These results indicate that cognitive improvements occurred specifically during the intervention period, reinforcing the efficacy of the treatment.

The GEE analysis, which jointly modeled data from all assessment points, demonstrated a robust and independent effect of the AI-driven intervention on global cognitive function. The primary outcome, MMSE, showed a highly significant improvement during active intervention periods compared to control periods (*p* < 0.001), even after stringent FDR correction. While secondary outcomes, such as DSF, TMT-A/B, and psychosocial scales, exhibited modest improvements, these changes did not reach statistical significance in the GEE model after multiple comparison adjustments (Table 3).

**Table 3:** Effects of ai-driven telerehabilitation: GEE analysis and crossover changes.

Outcome measures	Change during intervention	Change during control	GEE *p*-value	Adjusted *p*-value (FDR)
Primary outcome				
MMSE	3.0 [2.0, 4.0]	0.0 [0.0, 1.0]	**<0.001** ^*^	**<0.001** ^*^
Secondary outcomes				
DSF	0.1 [−0.2, 1.0]	0.0 [−0.1, 0.9]	0.548	0.856
DSB	0.1 [−0.1, 0.9]	0.0 [−0.1, 0.9]	0.746	0.856
TMT-A (sec)	−5.0 [−15.0, 3.0]	−1.0 [−9.0, 7.5]	0.215	0.682
TMT-B (sec)	−14.0 [−34.0, 3.0]	−5.0 [−20.0, 4.5]	0.303	0.682
CES-D	0.0 [−9.0, 2.0]	0.0 [−3.5, 0.5]	0.248	0.682
EQ-5D	0.01 [0.00, 0.05]	0.01 [−0.01, 0.05]	0.701	0.856
SES	1.0 [−1.5, 3.5]	1.5 [−1.5, 4.5]	0.761	0.856
S-IADL	0.0 [−0.5, 0.0]	−0.5 [−1.0, 0.0]	0.938	0.938

Values are presented as median [1st quartile, 3rd quartile] to account for the non-normal distribution of continuous variables. GEE p-value indicates the significance of the intervention effect derived from the Generalized Estimating Equations (GEE) model, adjusting for period and sequence effects. Adjusted p-value (FDR) denotes p-values corrected using the False Discovery Rate (FDR) method to account for multiple comparisons across all outcome measures.

MMSE, Mini-Mental State Examination; DSF, Digit Span Forward; DSB, Digit Span Backward; TMT, Trail Making Test; CES-D, Center for Epidemiologic Studies Depression Scale; EQ-5D, EuroQoL 5-Dimension; SES, Self-Efficacy Scale; S-IADL, Seoul-Instrumental Activities of Daily Living. ^*^Indicates statistical significance (*p* < 0.05).

To further assess treatment efficacy using a clinically meaningful cutoff, MMSE scores were dichotomized based on a threshold of 27 points. Participants scoring 26 or below were categorized as “fail,” and those scoring 27 or above were categorized as “success.” At baseline (T0), none of the participants achieved the “success” criterion (MMSE ≥27), reflecting uniform cognitive impairment across both groups. At T1, following the first intervention period, 93.9% of participants in Group AB (intervention group) reached the success threshold (*n* = 31/33), while none in Group BA (control group) did (*n* = 0/29). This group difference was statistically significant [χ^2^(1) = 54.49, *p* < 0.001]. By T2, after both groups had received the intervention, Group AB maintained high success rates (90.9%, 30/33), and Group BA showed substantial improvement, with 89.7% reaching MMSE ≥27 (26/29). The group difference at T2 was no longer statistically significant [χ^2^(1) = 0.03, *p* = 0.868]. These results confirm that the cognitive gains observed were primarily driven by the intervention and occurred specifically during the treatment phase, as expected in a delayed-treatment crossover design (Table 4).

**Table 4:** Success rates based on MMSE cutoff (≥27) at each time point.

Time point	Group AB (*n* = 33)	Group BA (*n* = 29)	Statistics (χ^2^)	*p*-value
Baseline (T0)	0 (0.0%)	0 (0.0%)	–	>0.999
Period 1 (T1)	31 (93.9%)	0 (0.0%)	54.49	<0.001^*^
Period 2 (T2)	30 (90.9%)	26 (89.7%)	0.03	0.868

Values are presented as number (%). Success was defined as an MMSE score of 27 or higher. Group AB, Intervention first, then no treatment; Group BA, No treatment first, then intervention. T0, baseline, T1, post-period 1, T2, post-period 2. ^*^Indicates statistical significance (*p* < 0.05).

### 3.3 System usability and user experience assessment

Table 5 summarizes usability outcomes across eight domains for both groups. Usability was assessed with a standardized questionnaire encompassing these domains. The average ratings for overall product quality and overall satisfaction were 4.6 ± 0.7 and 4.7 ± 0.5, respectively. Participants did not report any notable difficulties related to system usability, indicating favorable acceptance of the cognitive rehabilitation platform. Additionally, no significant adverse events leading to participant discontinuation occurred in either group.

**Table 5:** Descriptive Statistics of usability questionnaire responses.

Questionnaire items	Group AB (*n* = 33)	Group BA (*n* = 29)	Total (*n* = 62)
Equitable use and flexibility in use	4.3 ± 0.6	4.1 ± 0.8	4.2 ± 0.7
Simple and intuitive use	4.4 ± 0.6	4.2 ± 0.6	4.3 ± 0.6
Perceptible information	4.3 ± 0.7	4.2 ± 0.7	4.2 ± 0.7
Tolerance for error	4.2 ± 0.7	3.9 ± 0.8	4.1 ± 0.8
Low physical effort	4.4 ± 0.7	4.1 ± 0.8	4.3 ± 0.7
Size and space for user	4.8 ± 0.5	4.6 ± 0.6	4.7 ± 0.5
Overall product quality	4.6 ± 0.6	4.5 ± 0.5	4.6 ± 0.5
Overall satisfaction	4.6 ± 0.7	4.7 ± 0.5	4.7 ± 0.6

Values are presented as mean ± SD. Group AB, Intervention first, then no treatment; Group BA, No treatment first, then intervention.

## 4 Discussion

### 4.1 Summary of the principle findings

The findings confirm that the self-guided, AI-driven telerehabilitation platform provides a highly feasible and effective non-pharmacological strategy for managing cognitive decline in MCI. Evaluation at T1 demonstrated that participants undergoing the active intervention showed statistically significant superior gains in MMSE scores compared to the control group (no intervention). This effect was further substantiated by the crossover findings, where the control-first group exhibited a significant increase in MMSE scores immediately following their active intervention phase (T2). Crucially, the favorable safety profile, characterized by high participant satisfaction and the complete absence of serious adverse events, supports its potential for widespread integration into clinical care pathways.

Our findings align with and extend prior evidence suggesting that computerized cognitive rehabilitation can enhance several cognitive domains in individuals with MCI (16, 17). A systematic reviews reported improvement in memory, working memory, attention, processing speed, and executive function following computer-based cognitive interventions, while showing inconsistent effects on global cognition and language abilities (18).

In contrast to these mixed findings, the present study demonstrated robust improvements in global cognition, as shown by consistent MMSE gains across both intervention periods. The consistent improvements observed across both groups suggest that the system's ability to autonomously adjust difficulty and personalize content allowed participants to effectively engage in cognitive training without continuous therapist supervision, thereby facilitating meaningful cognitive gains in a self-guided home setting. While previous research has confirmed the general efficacy of cognitive telerehabilitation, our study extends these findings by demonstrating the clinical value of AI-enabled adaptive learning in a home-based setting. Therefore, our findings highlight an important advancement: the integration of AI-enabled adaptive learning within a home-based telerehabilitation framework.

The results are also consistent with previous work demonstrating that telerehabilitation-based cognitive interventions can achieve outcomes comparable to face-to-face therapy across various neurological conditions. A meta-analysis showed that telerehabilitation yields cognitive benefits on par with in-person cognitive rehabilitation and may even confer advantages in working memory and executive functions (19). Similarly, other authors have reported that remotely delivered cognitive treatment improves multiple cognitive domains with efficacy equivalent to conventional therapy formats. Our study reinforces these observations by demonstrating that an AI-enhanced telerehabilitation platform can deliver clinically meaningful cognitive outcomes even without direct therapist supervision (20).

The consistent pattern of MMSE improvement during intervention phases, coupled with the absence of gains during control periods, highlights the therapeutic specificity of the AI-driven computerized cognitive rehabilitation program. While secondary outcomes such as mood, self-efficacy, and health-related quality of life improved modestly over time, these changes did not differ between intervention and control phases, suggesting a nonspecific engagement or social participation effect rather than a treatment-related improvement.

The minimal change observed in executive function, attention, and working memory tests may reflect the relatively short intervention duration or ceiling effects inherent to the cognitively healthy spectrum of MCI participants. Notably, the baseline MMSE scores of our participants averaged 26 points, positioning them at the uppermost limit of the inclusion criteria (18–26 points). This high baseline cognitive status likely restricted the window for further measurable improvement in specific, high-difficulty cognitive domains. In this context, the MMSE may have functioned as a more robust indicator of global clinical change, as its composite score can reflect the cumulative impact of subtle improvements across multiple cognitive domains that do not reach statistical significance individually.

High usability ratings across all domains indicate that older adults with MCI could engage with the system comfortably and with minimal operational difficulty. The absence of device-related adverse events and the excellent adherence observed throughout the study further support the feasibility of implementing AI-based cognitive telerehabilitation in community settings. These findings are particularly relevant given persistent barriers to clinic-based cognitive rehabilitation, including mobility limitations, caregiver dependence, and resource constraints.

Importantly, the observed cognitive improvements were accompanied by high adherence and sustained engagement with the AI-driven training program. As shown in Table 5, participants completed a substantial proportion of the prescribed sessions, with consistent training frequency and duration across intervention periods. These findings suggest that the home-based AI-driven computerized cognitive rehabilitation system was feasible and acceptable for older adults with MCI, supporting its potential scalability in real-world settings.

### 4.2 Limitations and future directions

Several limitations should be acknowledged. First, a potential selection bias may have influenced the study outcomes. The participants had a mean baseline MMSE score of approximately 26 points, placing them at the uppermost limit of the MCI spectrum. This high baseline cognitive status suggests that the sample predominantly consisted of “high-functioning” MCI individuals, which likely introduced a ceiling effect. Consequently, this proximity to the threshold for cognitive success (MMSE ≥27) may have restricted the window for observable improvement and constrained the clinical interpretability of the results. Even statistically significant gains may reflect modest absolute changes that do not necessarily translate into substantial functional benefits. Second, measurement bias related to the assessment tools must be considered. While global cognitive measures like the MMSE showed significant gains, domain-specific neuropsychological tests demonstrated limited sensitivity. This discrepancy raises the possibility of a “measurement gap,” where standard tests may not be granular enough to capture the specific cognitive shifts induced by AI-driven training within a short timeframe. Furthermore, practice effects due to repeated testing—particularly with the MMSE—cannot be entirely ruled out and may have partially contributed to score improvements independent of the intervention. Therefore, the observed cognitive gains should be interpreted with caution as they may partially reflect increased test familiarity. Future studies should employ alternate test forms to better isolate the therapeutic effects. Third, the intervention duration was relatively short, leaving the long-term durability of these cognitive gains unknown. Fourth, the sample comprised predominantly older women, which limits the generalizability of the findings to the broader, more diverse MCI population, including different genders and socioeconomic backgrounds.

Future investigations should aim to strengthen the clinical validity, mechanistic understanding, and translational applicability of AI-driven cognitive training in patients with MCI. First, larger multicenter randomized controlled trials with extended follow-up durations are required to determine the long-term sustainability of training-induced cognitive improvements and to assess whether such gains translate into meaningful clinical outcomes, including delayed progression to dementia, maintenance of functional independence, and preservation of quality of life. Second, future studies should enroll participants with a wider spectrum of baseline cognitive impairment and stratify cohorts according to MCI subtypes (amnestic vs. non-amnestic; single- vs. multiple-domain MCI). This stratification will allow identification of patient subgroups that derive the greatest benefit and support personalized, stage-specific application of AI-based cognitive rehabilitation. Finally, methodological refinements—such as the use of alternate test forms, longer inter-assessment intervals, and statistical modeling of practice effects—are needed to better isolate intervention-related cognitive improvements from repeated testing effects.

## 5 Conclusion

This study provides compelling evidence that AI-driven telerehabilitation can produce meaningful short-term improvements in global cognitive function among individuals with MCI. Given the scalability, accessibility, and minimal supervision requirements of AI-based telerehabilitation, such interventions may serve as a practical adjunct or alternative to conventional clinic-based cognitive therapy, particularly in aging societies with limited rehabilitative resources.

## Data Availability

The raw data supporting the conclusions of this article will be made available by the authors, without undue reservation.

## References

[1] Giri B Singh DB Chattu VK. Aging population in South Korea: burden or opportunity? Int J Surg Glob Health. (2024) 7:e0517. doi: 10.1097/GH9.0000000000000517

[2] CampbellJ LavoieL FarraiaM HuelinR ZhangQ Tahami MonfaredAA . Quality of life in mild cognitive impairment and mild dementia associated with alzheimer's disease: a systematic review. Neurol Ther. (2025) 14:7–26. doi: 10.1007/s40120-024-00676-939489884 PMC11762030

[3] GopalakrishnanP TiwariS NagarajaR KrishnanG. Quality of life in persons with mild cognitive impairment: a systematic review and meta-analysis. Dement Neuropsychol. (2024) 18:e20230093. doi: 10.1590/1980-5764-dn-2023-009339193465 PMC11348882

[4] Mitchell AJ Shiri-Feshki M. Rate of progression of mild cognitive impairment to dementia—meta-analysis of 41 robust inception cohort studies. Acta Psychiatr Scand. (2009) 119:252–65. doi: 10.1111/j.1600-0447.2008.01326.x 19236314

[5] ChenY QianX ZhangY SuW HuangY WangX . Prediction models for conversion from mild cognitive impairment to Alzheimer's disease: a systematic review and meta-analysis. Front Aging Neurosci. (2022) 14:910747. doi: 10.3389/fnagi.2022.84038635493941 PMC9049273

[6] Hong YJ Jang EH Hwang J Roh JH Lee JH. The efficacy of cognitive intervention programs for mild cognitive impairment: a systematic review. Curr Alzheimer Res. (2015) 12:527–42. doi: 10.2174/1567205012666150530201636 26027815

[7] Bahar-FuchsA ClareL WoodsB. Cognitive training and cognitive rehabilitation for mild to moderate Alzheimer's disease and vascular dementia. Cochrane Database Syst Rev. (2013) 6:CD003260. doi: 10.1002/14651858.CD003260.pub223740535 PMC7144738

[8] MaggioMG De BartoloD CalabròRS CiancarelliI CerasaA ToninP . Computer-assisted cognitive rehabilitation in neurological patients: state-of-art and future perspectives. Front Neurol. (2023) 14:1255319. doi: 10.3389/fneur.2023.125531937854065 PMC10580980

[9] Nie P Liu F Lin S Guo J Chen X Chen S . The effects of computer-assisted cognitive rehabilitation on cognitive impairment after stroke: a systematic review and meta-analysis. J Clin Nurs. (2022) 31:1136–48. doi: 10.1111/jocn.16030 34459041

[10] KimS ParkSW JeongT KangMS KimDY. AI-driven cognitive telerehabilitation for stroke: a randomized controlled trial. Front Neurol. (2025) 16:1636017. doi: 10.3389/fneur.2025.163601740895105 PMC12391089

[11] Hill NT Mowszowski L Naismith SL Chadwick VL Valenzuela M Lampit A. Computerized cognitive training in older adults with mild cognitive impairment or dementia: a systematic review and meta-analysis. Am J Psychiatry. (2017) 174:329–40. doi: 10.1176/appi.ajp.2016.16030360 27838936

[12] PramukaM van RoosmalenL. Telerehabilitation technologies: accessibility and usability. Int J Telerehabil. (2009) 1:85–98. doi: 10.5195/ijt.2009.601625945165 PMC4296785

[13] KimS KimDY ParkSW JeonN JeongT KangMS . Artificial intelligence-guided mobile telerehabilitation for individuals with cognitive impairment: a feasibility study. Ann Rehabil Med. (2025) 49:371–80. doi: 10.5535/arm.25006041492723 PMC12771165

[14] BaekMJ ParkYH KimS. Comparison between the mini-mental state examination and the mini-mental state examination-2 in patients with mild cognitive impairment and Alzheimer's disease. J Korean Med Sci. (2025) 40:e235. doi: 10.3346/jkms.2025.40.e23540955614 PMC12437240

[15] KimM HeoD KimS LeeY KimYS SungW . Cognitive impairment screening test in Korea as a screening tool for dementia: the correlation study of subtest scores with Korean version of the mini mental state examination 2nd edition. Dement Neurocogn Disord. (2025) 24:126–34. doi: 10.12779/dnd.2025.24.2.12640321439 PMC12046244

[16] KlimovaB MaresovaP. Computer-based training programs for older people with mild cognitive impairment and/or dementia. Front Hum Neurosci. (2017) 11:262. doi: 10.3389/fnhum.2017.0026228559806 PMC5432561

[17] LiR GengJ YangR GeY HeskethT. Effectiveness of cognitive training in delaying cognitive function decline in people with mild cognitive impairment: systematic review and meta-analysis. J Med Internet Res. (2022) 24:e38624. doi: 10.2196/3862436301590 PMC9650579

[18] ZuschneggJ SchobererD HäusslA HerzogSA RusseggerS PloderK . Effectiveness of computer-based interventions for community-dwelling people with cognitive decline: a systematic review with meta-analyses. BMC Geriatr. (2023) 23:229. doi: 10.1186/s12877-023-03941-y37041494 PMC10091663

[19] CaccianteL della PietàC RutkowskiS CieślikB Szczepańska-GierachaJ AgostiniM . Cognitive telerehabilitation in neurological patients: systematic review and meta-analysis. Neurol Sci. (2022) 43:847–62. doi: 10.1007/s10072-021-05770-634822030 PMC8613517

[20] Maggio MG De Luca R Manuli A Calabrò RS. The five ‘W' of cognitive telerehabilitation in the COVID-19 era. Expert Rev Med Devices. (2020) 17:473–5. doi: 10.1080/17434440.2020.1776607 32476504

