# Supplementary material for: AI-driven telerehabilitation for older adults with mild cognitive impairment: a randomized controlled trial
Source: Front Neurol. 2026 Apr 28;17:1813694. doi: 10.3389/fneur.2026.1813694 (PMC13160745; doi:10.3389/fneur.2026.1813694)
Supplement: Supplementary file 1 [file Table_1.docx]

Supplementary Material

# Supplementary Material 1. Composition of Cognitive Training Modules and Specific Tasks in Zenicog®

| Domain | Subdomain | Task |
| --- | --- | --- |
| **Cognitive Domain** | **Attention** | Identifying identical figures |
|  |  | Simple reaction task |
|  |  | Pattern replication |
|  |  | Number counting |
|  |  | Arithmetic calculation |
|  |  | Target detection (colored balloons) |
|  |  | Target cancellation (balloon popping) |
|  |  | Visual search (hidden picture task) |
|  |  | Symmetry construction |
|  |  | Body part identification (left/right) |
|  |  | Number search (telephone number identification) |
|  |  | Selective attention task (target among distractors) |
|  | **Memory** | Digit span (number recall) |
|  |  | Visual memory (figure recall) |
|  |  | Auditory memory (sound recall) |
|  |  | Spatial memory (location recall) |
|  |  | Verbal memory (word recall) |
|  |  | Code-breaking task (symbol decoding) |
|  |  | Feature memory (attribute recall) |
|  |  | Functional memory task (shopping simulation) |
|  | **Executive Function** | Odd-one-out task |
|  |  | Categorization |
|  |  | Arithmetic problem generation |
|  |  | Visuospatial puzzle |
|  |  | Functional calculation (making change) |
|  |  | Word puzzle |
|  |  | Tower of Hanoi |
| **Language Domain** | **Auditory Comprehension** | Yes/No judgment (picture-based) |
|  |  | Yes/No judgment (word-based) |
|  |  | Auditory word recognition |
|  |  | Picture selection |
|  |  | Auditory sequencing (word ordering) |
|  |  | Semantic–picture matching |
|  |  | Following verbal instructions |
|  | **Speaking**  **(Expressive Language)** | Word repetition |
|  |  | Prosody training |
|  |  | Automatic speech production |
|  |  | Nonword repetition |
|  |  | Confrontation naming |
|  |  | Sentence repetition |
|  |  | Verbal response to description |
|  |  | Digit repetition |
|  |  | Word association |
|  |  | Word combination (phrase construction) |
|  |  | Verbal description |
|  | **Reading** | Picture–word matching |
|  |  | Word reading |
|  |  | Word–picture matching |
|  |  | Picture-based response selection |
|  |  | Cloze task (fill-in-the-blank) |
|  |  | Nonword reading |
|  |  | Number reading |
|  |  | Semantic–picture matching |
|  |  | Semantic–word matching |
|  |  | Sentence reading |
|  |  | Following written instructions |
|  | **Writing** | Copying |
|  |  | Fill-in-the-blank writing |
|  |  | Written naming (picture-based) |
|  |  | Word combination (written expression) |
|  |  | Dictation |

**Supplementary Material 2. AI/ML Components Implemented in Zenicog****®**

The system can be characterized as a hybrid AI architecture, combining supervised machine learning models with adaptive algorithmic components for personalized cognitive training and assessment.

**1. Supervised Machine Learning for Cognitive Assessment Prediction**

Zenicog® incorporates supervised learning models to estimate cognitive status based on user interaction data.

A regression model predicts continuous cognitive assessment scores (e.g., MMSE) from longitudinal training performance.

• A classification model estimates the probability distribution across multiple cognitive severity levels.

Both models are trained on structured features derived from user training history, including:

• performance accuracy across training types

• progression of task difficulty

• aggregated cognitive function indicators mapped to standardized functional domains (e.g., ICF framework)

Feature extraction is performed over a configurable temporal window of recent training sessions, enabling the models to capture short-term cognitive trends.

**2. Multi-Criteria Adaptive Recommendation Mechanism**

The recommendation system is not based on simple rule-based logic but instead uses a multi-factor scoring framework that integrates:

• alignment between task difficulty and estimated user ability

• diversity and recency of training experiences

• user-specific response and problem-solving time profiles

• session-level adaptive adjustments

These factors are combined using empirically tuned weighting schemes to produce a composite relevance score for candidate training tasks. This approach enables context-aware and personalized task selection, going beyond deterministic or static recommendation rules.

**3. Adaptive Difficulty Adjustment**

The system continuously adapts task difficulty based on recent user performance.

• A recent-performance window is used to estimate the current ability level

• Task difficulty and time constraints are dynamically adjusted relative to target performance ranges

• A hierarchical fallback strategy is applied in cold-start or sparse-data scenarios, incorporating similarity, domain proximity, and population-level priors

This mechanism ensures that training remains appropriately challenging while avoiding under- or over-difficulty.

**4. Probabilistic Task Selection**

To balance personalization and exploration, Zenicog® employs probabilistic sampling strategies, including:

• inverse-frequency weighting to encourage exposure to underrepresented tasks

• randomized selection under weighted constraints to maintain variability

This prevents repetitive task assignment while preserving individualized relevance.

**5. Domain-Aware Knowledge Transfer**

The system incorporates domain-level generalization using mappings between training tasks and standardized cognitive function categories.

Performance in one task influences recommendations for related tasks within the same or adjacent functional domains

• This enables a form of cognitive domain transfer, supporting broader generalization of training effects

**6. Hierarchical Cognitive Scoring**

Zenicog® computes cognitive performance using a hierarchical aggregation framework:

• task-level performance is adjusted using behavioral indicators such as response time and solving time

• higher-level scores are computed through aggregation across training types and cognitive domains

• final scores represent integrated cognitive function across multiple domains

This structure improves robustness by reducing noise from individual task variability.

**Clinical and Methodological Justification**

These design choices are consistent with established principles in cognitive rehabilitation, where adaptive difficulty calibration, training variability, and domain generalization are known to influence intervention effectiveness. Model behavior and recommendation dynamics were evaluated through internal validation and real-world usage data to ensure stability, consistency, and clinical plausibility across diverse user populations.

**Clarification on “AI-driven” Terminology**

While the recommendation component is not based on deep neural networks, it incorporates data-driven personalization, probabilistic decision-making, and adaptive optimization mechanisms. Therefore, the term “AI-driven” is used in accordance with current clinical and regulatory perspectives (e.g., WHO digital health frameworks and FDA guidance on AI/ML-based software as a medical device), where such adaptive systems are considered valid AI-based interventions.

| **Supplementary Material 3. Descriptive Statistics of Outcome Measures by Time Point and Group** | | | | | | | | | |
| --- | --- | --- | --- | --- | --- | --- | --- | --- | --- |
|  | MMSE | DSF | DSB | TMT-A | TMT-B | CES-D | EQ-5D | SES | S-IADL |
| Group AB (n=33) |  |  |  |  |  |  |  |  |  |
| - T0 | 26.0 [25.0, 26.0] | 5.1 [4.2, 6.1] | 3.3 [3.1, 4.1] | 42.0 [35.0, 54.0] | 108.0 [78.0, 171.0] | 5.0 [1.0, 18.0] | 0.81 [0.75, 0.85] | 29.0 [27.0, 32.0] | 2.0 [1.0, 3.0] |
| - T1 | 28.0 [27.0, 29.0] | 5.2 [5.1, 6.1] | 3.3 [3.2, 4.1] | 33.0 [31.0, 46.0] | 86.0 [67.0, 130.0] | 4.0 [1.0, 8.0] | 0.82 [0.80, 0.86] | 29.0 [27.0, 30.0] | 2.0 [1.0, 2.0] |
| - T2 | 29.0 [28.0, 30.0] | 5.3 [5.1, 6.1] | 4.1 [3.2, 4.2] | 35.0 [26.0, 48.0] | 84.0 [62.0, 117.0] | 4.0 [2.0, 9.0] | 0.82 [0.78, 0.86] | 30.0 [26.0, 34.0] | 1.0 [0.0, 2.0] |
| Group BA (n=29) |  |  |  |  |  |  |  |  |  |
| - T0 | 26.0 [25.0, 26.0] | 5.1 [4.3, 5.3] | 3.3 [3.1, 4.1] | 34.0 [30.0, 50.0] | 103.0 [77.0, 162.0] | 6.0 [3.0, 13.0] | 0.83 [0.78, 0.86] | 29.0 [27.0, 31.0] | 1.0 [0.0, 2.0] |
| - T1 | 26.0 [26.0, 26.0] | 5.2 [4.2, 6.2] | 4.1 [3.1, 4.1] | 35.0 [28.0, 44.0] | 110.0 [76.0, 146.0] | 5.0 [2.0, 9.0] | 0.86 [0.82, 0.86] | 30.0 [30.0, 34.0] | 1.0 [0.0, 2.0] |
| - T2 | 29.0 [28.0, 29.0] | 5.2 [5.1, 6.1] | 4.1 [3.2, 4.2] | 31.0 [26.0, 39.0] | 92.0 [65.0, 118.0] | 2.0 [1.0, 8.0] | 0.85 [0.82, 0.90] | 30.0 [29.0, 36.0] | 1.0 [0.0, 1.0] |

Note. Values are presented as median [1st quartile, 3rd quartile]. Group AB = Intervention first, then no treatment; Group BA = No treatment first, then intervention. T0 = baseline, T1 = post-period 1, T2 = post-period 2.

MMSE = Mini-Mental State Examination; DSF = Digit Span Forward; DSB = Digit Span Backward; TMT = Trail Making Test; CES-D = Center for Epidemiologic Studies Depression Scale; EQ-5D = EuroQoL 5-Dimension; SES = Self-Efficacy Scale; S-IADL = Seoul-Instrumental Activities of Daily Living.
